# 1894. Delay in TB care before and during the COVID-19 pandemic, Dushanbe, Tajikistan, 2022

**DOI:** 10.1093/ofid/ofad500.1722

**Published:** 2023-11-27

**Authors:** Rajabali Sharifov, Dilyara Nabirova, Zulfiya Haybullo Tilloeva, Sanam Zikriyarova, Salomudin Yusufi, Navruz Jafarov, Roberta Horth

**Affiliations:** Central Asia Region FETP, Dushanbe, Dushanbe, Tajikistan; CDC Central Asia office, Almaty, Almaty, Kazakhstan; City Desinfection station, Dushanbe, Republic of Tajikistan, Dushanbe, Dushanbe, Tajikistan; Kazakh National Medical University named after S.D. Asfendiyarov;, Almaty, Almaty, Kazakhstan; Ministry of Health, Dushanbe, Dushanbe, Tajikistan; Ministry of Health, Dushanbe, Dushanbe, Tajikistan; US Centers for Disease Control and Prevention, Dulles, Virginia

## Abstract

**Background:**

Delays in TB treatment prolong the period of infectivity and are associated with drug resistance. Tajikistan has a high and increasing burden of multidrug-resistant tuberculosis (incidence of 28/100,000 in 2021). Many new cases present with severe disease, indicating delayed care. The COVID-19 pandemic further threatened to undo progress.

**Methods:**

We conducted a retrospective cohort study of people >15 years old newly diagnosed with pulmonary TB in Dushanbe in 2019-2021. We conducted face-to-face interviews using structured questionnaires and abstracted data from medical records. We defined patient-delay as >14 days between TB symptom onset and initial visit, and provider-delay as >3 days from initial visit to treatment initiation. We ran multivariable Poisson regressions to estimate variables associated with presentation-delay and provider-delay.

**Results:**

From 2019-21, 472 people were newly diagnosed with pulmonary TB. Of these, 82% had delayed treatment, 49% were male, 61% had lung tissue decay, 11% had diabetes, and 4% had HIV. Additionally, 42% had been diagnosed in 2019. The proportion with patient-delay was similar before and during the pandemic (83% vs. 82%, respectively, p=0.8). However, the proportion with provider-delay was higher before than during the pandemic (44% vs. 34%, p=0.02). There was no statistical difference in median days of patient-delay before (60 days; interquartile range [IQR]: 15–541) and after (60 days; IQR: 15–360, p=0.6) the pandemic, nor in median days of provider-delay before (7 days; IQR: 4–336) and after (7 days; IQR: 4–225, p=0.6) the pandemic. In multivariable analysis, the COVID-19 pandemic was not associated patient-delay (adjusted risk ratio and 95% confidence interval=1.00; 0.96-1.04). Patient-delay was significantly higher in people who ignored disease symptoms (1.41; 1.20-1.65) and thought services were expensive (1.10; 1.01-1.20) even though they are free of charge (Table 1).

**Figure:**
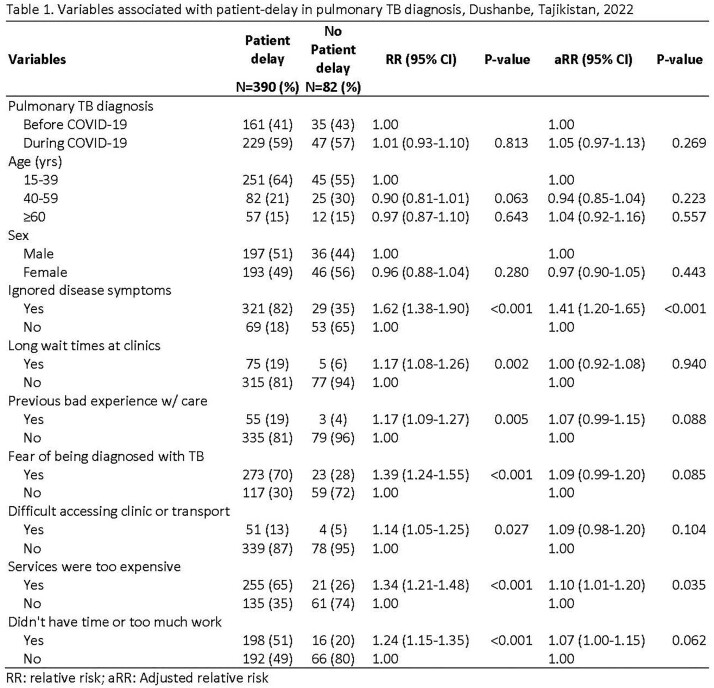

**Conclusion:**

Contrary to expected, presentation-delay for TB diagnosis did not differ before and during the COVID-19 pandemic, and provider-delay was lower. Increased vigilance of respiratory diseases and differential diagnosis for SARS-CoV-2 may have resulted in timelier TB diagnosis. However, interventions to further reduce delays are still needed.

**Disclosures:**

**All Authors**: No reported disclosures

